# Tratamiento ortopédico prequirúrgico con retractor nasal modificación Hinostroza en fisura labiopalatina unilateral completa. Reporte de caso

**DOI:** 10.21142/2523-2754-0902-2021-065

**Published:** 2021-06-21

**Authors:** Manuel Hinostroza-Flores, Guido Alberto Perona-Miguel de Priego, Jennifer Loo-Valle

**Affiliations:** 1 Maestría de Odontopediatría, Universidad Científica del Sur. Lima, Perú. odonto21@hotmail.com, gperona@cientifica.edu.pe Universidad Científica del Sur Maestría de Odontopediatría Universidad Científica del Sur Lima Peru odonto21@hotmail.com gperona@cientifica.edu.pe; 2 Especialidad de Odontopediatría, Universidad Científica del Sur. Lima, Perú. drajenniferloo@gmail.com Universidad Científica del Sur Especialidad de Odontopediatría Universidad Científica del Sur Lima Peru drajenniferloo@gmail.com

**Keywords:** labio fisurado, paladar hendido, ortopedia infantil prequirúrgica, cleft lip, cleft palate, presurgical infant orthopedics

## Abstract

La fisura labiopalatina unilateral completa afecta la región del tercio medio facial y los individuos que nacen con esta condición presentan estructuras alteradas en el lado donde se desarrolló la malformación. El objetivo del presente reporte de caso es presentar una alternativa de ortopedia prequirúrgica para pacientes con fisura labiopalatina unilateral completa en una infante de sexo femenino de 1 mes y 5 días de edad con el diagnóstico de fisura labiopalatina unilateral izquierda completa. Se colocó el retractor nasal modificación Hinostroza, que permite obtener simetría de la estructura nasal afectada con respecto al lado no afectado; por sus buenos resultados, bajo costo y fácil manejo debe ser considerada una alternativa de tratamiento en neonatos nacidos con fisura labiopalatina unilateral completa.

## Abstract (en)

Complete unilateral palatine fissure affects the region of the middle facial third and individuals born with this condition have altered structures on the side where the malformation developed. The objective of the present case report was to describe a pre-surgical orthopedics alternative in a female infant of 1 month 5 days of age with complete unilateral palatine fissure diagnosed with complete unilateral left palatine fissure. The Hinostroza nasal retractor modification was placed, allowing symmetry to the affected nasal structure with respect to the unaffected side. This procedure provides good results, low cost and easy handling and should be considered as an alternative treatment in neonates born with complete unilateral palatine fissure.

## INTRODUCCIÓN

La fisura labiopalatina es la malformación congénita más frecuente a nivel mundial. En el Perú, se reporta una incidencia de 0,8 a 1,7 por cada 1000 nacidos vivos ^1^. No se conoce exactamente la etiología, pero la literatura menciona que es multifactorial, ya que intervienen factores genéticos y ambientales ^2^.

Durante el periodo embrionario, los procesos nasales mediales se fusionan con los procesos maxilares para formar el labio superior y el paladar primario a las 6 semanas de vida intrauterina ^3^. A partir de la semana 8 hasta la semana 12, los procesos palatinos, que son las prolongaciones horizontales del proceso maxilar, se orientan en dirección a la línea media y se fusionan entre sí para formar el paladar secundario, el cual, a su vez, se fusiona hacia adelante con el paladar primario y hacia arriba con el septum nasal. Cuando se produce una falta de fusión de estos procesos da como resultado la fisura labiopalatina unilateral o bilateral, lo que afecta las funciones del sistema estomatognático ^4^. 

En la fisura labiopalatina unilateral completa, el maxilar y las estructuras que de él derivan están alteradas debido a la presencia de un maxilar completamente desarticulado y se observa una rotación vestibular del segmento mayor y una rotación palatina del segmento menor, esto causa desplazamiento en grados variables de la punta nasal, tabique nasal, columela y cartílago nasal hacia la zona donde se ha desarrollado el defecto ^5^^,^^6^. 

La ortopedia prequirúrgica es el tratamiento que emplea fuerzas ortopédicas para posicionar las estructuras maxilares alteradas debido a una deformidad ocasionada por la fisura labiopalatina ^7^. Los aparatos ortopédicos prequirúrgicos se desarrollaron para corregir las estructuras alteradas y guiar el crecimiento ^8^.

Actualmente, se conocen muchos beneficios logrados con el uso de aparatos ortopédicos prequirúrgicos, como la reducción en el ancho de la fisura palatina, el mejor desarrollo del arco maxilar, la mejora de la anatomía del cartílago nasal afectado, y las mejoras en la alimentación, el habla, la audición y el lenguaje ^7^^,^^10^.

El retractor nasal es un aparato ortopédico prequirúrgico que está indicado en neonatos nacidos con fisura labiopalatina unilateral completa. Por tal motivo, es posible corregir la estructura del cartílago nasal, alinear la columnela y la punta nasal ^12^^-^^14^. De esa manera, se puede brindar las condiciones adecuadas al neonato para recibir cirugías primarias de labio y del paladar completamente seguras ^15^^,^^16^. 

## REPORTE DE CASO

**Figura 1:**
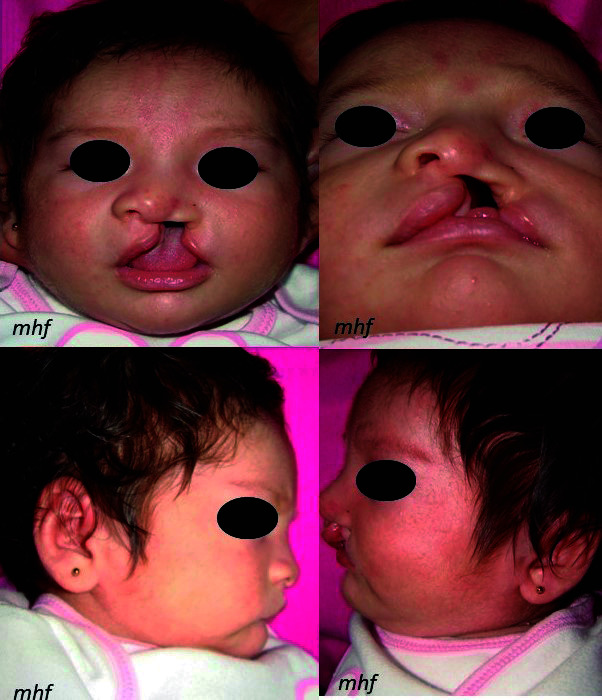
a) Foto frontal; b) Foto submentonial; c) Foto lateral derecha; d) Foto lateral izquierda

Neonato, sexo femenino, de 1 mes y 15 días de nacida, con buen estado de salud general, es llevada por la madre a consulta para tratamiento prequirúrgico por presentar fisura labiopalatina unilateral completa. Tras el examen clínico, se observa que presenta fisura labiopalatina completa unilateral izquierda, según la clasificación de Stark. Por las características clínicas presentes, se indicó el tratamiento ortopédico prequirúrgico con un retractor nasal modificación Hinostroza. Asimismo, se tomó un registro de fotografías iniciales (Figura 1).

**Figura 2:**
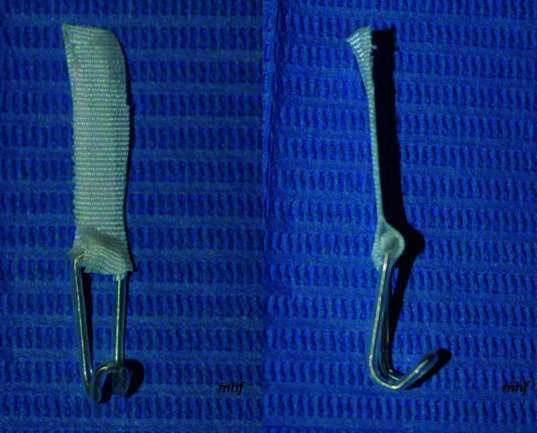
Retractor nasal modificación Hinostroza: **a)** Vista frontal; **b)** Vista lateral

El retractor nasal se confeccionó con un alambre de acero inoxidable número 0,6 mm, de 10 centímetros de longitud, con el mismo largo para el protector de alambre, y se realizó 6 dobleces con el alicate 139, con lo que se le dio la forma de un retractor; en el extremo superior, para unir los dos extremos terminales, se utilizó el Durapore® (3M) (Figura 2).

**Figura 3:**
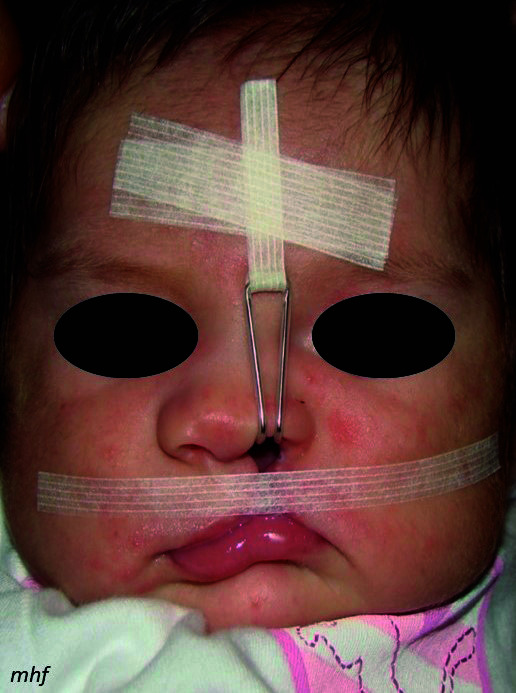
Colocación de retractor nasal (modificación Hinostroza) y Steri-Strip subnasal

**Figura 4:**
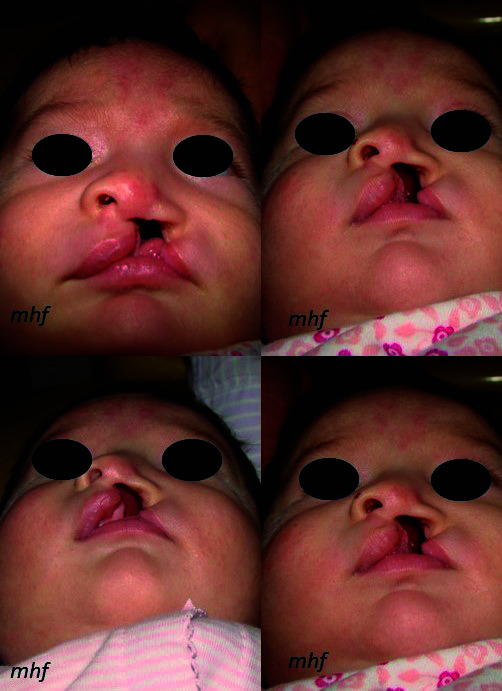
Controles del procedimiento

Posteriormente, se insertó el retractor nasal modificación Hinostroza en el ala nasal afectada usando la cinta Durapore® (3M). Se traccionó aplicando una fuerza de 1 onza hacia la región frontal, en una dirección paralela a la línea media facial, y se fijó con una cinta Micropore® (3M) en la región frontal (Figura 3). El Steri-Strip® (3M) subnasal se coloca por debajo de la punta nasal, desde el ángulo cantal externo derecho al izquierdo, con la finalidad de reducir el ancho de la fisura. Los controles se efectuaron cada 15 días hasta el día de la cirugía (Figura 4). 

## DISCUSIÓN

La confección del retractor nasal modificación Hinostroza se inspiró en los estudios realizados por Avni Abdiu *et al.* En este caso, se confeccionó un elevador nasal hecho de alambre y recubierto, en la misma cita, tomando la medida personalizada para cada paciente ^13^. Heravi *et al.* confeccionaron un elevador nasal con características diferentes, para lo cual utilizaron un alambre de acero número 0,7 mm y realizaron un doblez en cada extremo. En uno de los extremos colocaron una resina acrílica y en el otro, en forma de gancho, un elástico de 5/16 pulgadas para realizar la tracción hacia la región frontal ^17^.

Monasterio *et al.* realizaron un elevador nasal de un clip forrado con una cinta teflón y colocaron en un extremo un elástico de 5/16 pulgadas para aplicar la fuerza de tracción hacia la región frontal ^14^. Para evitar la placa intraoral, se sustituyó la cinta de papel por una banda elástica conocida como DynaCleft® (Canica Design Inc.). El inconveniente del uso de esta banda elástica junto con el elevador nasal es su alto costo.

En el presente reporte de caso, utilizamos una cinta Durapore® (3M) para realizar la tracción del retractor nasal hacia la región frontal y el Steri-Strip® colocado en la región subnasal para aproximar los segmentos maxilares y así reducir el ancho de la fisura, lo cual resultó una buena alternativa y de muy bajo costo.

Vinson ^8^ realizó un estudio en recién nacidos con fisura labiopalatina unilateral completa para comparar el uso de 2 métodos de ortopedia prequirúrgica: el Dynacleft® y el moldeador nasoalveolar (NAM). Su investigación demostró que el sistema Dynacleft® es un método adecuado para reducir el tamaño de la fisura y tiene bajo costo. 

**Figura 5:**
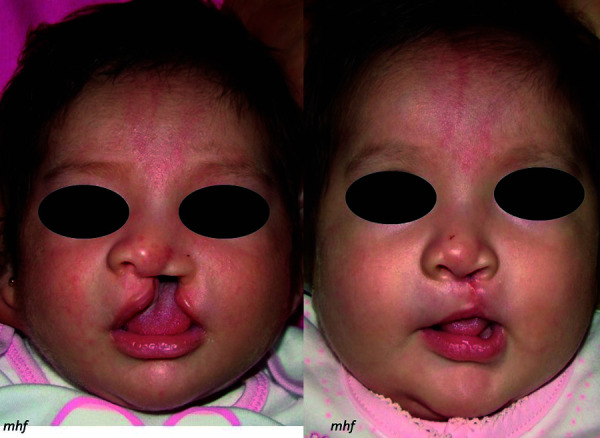
a) Antes del tratamiento; b) Después del tratamiento

Una mejor opción de ortopedia prequirúrgica que proponemos es el retractor nasal modificación Hinostroza junto con Steri-Strip® (3M), cuya principal ventaja es su fácil y rápida confección, mientras que otros sistemas, como el Dynacleft®, son de mayor costo. Por otra parte, el retractor nasal modificación Hinostroza comparado con el stend nasal del moldeador nasoalveolar (NAM) realiza las mismas funciones, como moldear la curvatura del cartílago nasal, alinear la columela y la punta nasal, lo que, en general, mejora la estructura nasal afectada ^5^^,^^16^^,^^18^^-^^20^ (Figura 5). La diferencia radica en que el retractor nasal es menos invasivo, tiene menor riesgo de obstrucción nasal, reduce el riesgo de lesiones en la mucosa nasal, es de fácil confección, tiene un bajo costo y fácil manejo, y sobre todo se elimina la toma de impresión del recién nacido. Además, brindamos indicaciones a los padres acerca de cómo instalar y retirar el retractor nasal, para que ellos puedan cambiar la cinta, realizar la limpieza del retractor nasal y colocarlo nuevamente. 

## CONCLUSIONES

El tratamiento ortopédico prequirúrgico con el uso del retractor nasal modificación Hinostroza permite obtener adecuados resultados estéticos en el cartílago nasal del lado afectado por la malformación, la alineación adecuada de la columela y la punta nasal. Por esto, debe ser considerado una excelente alternativa de ortopedia prequirúrgica, por sus beneficios, su bajo costo y fácil manejo para tratar a pacientes con fisura labiopalatina unilateral completa.

## Fuente de financiamiento:

Autofinanciado
